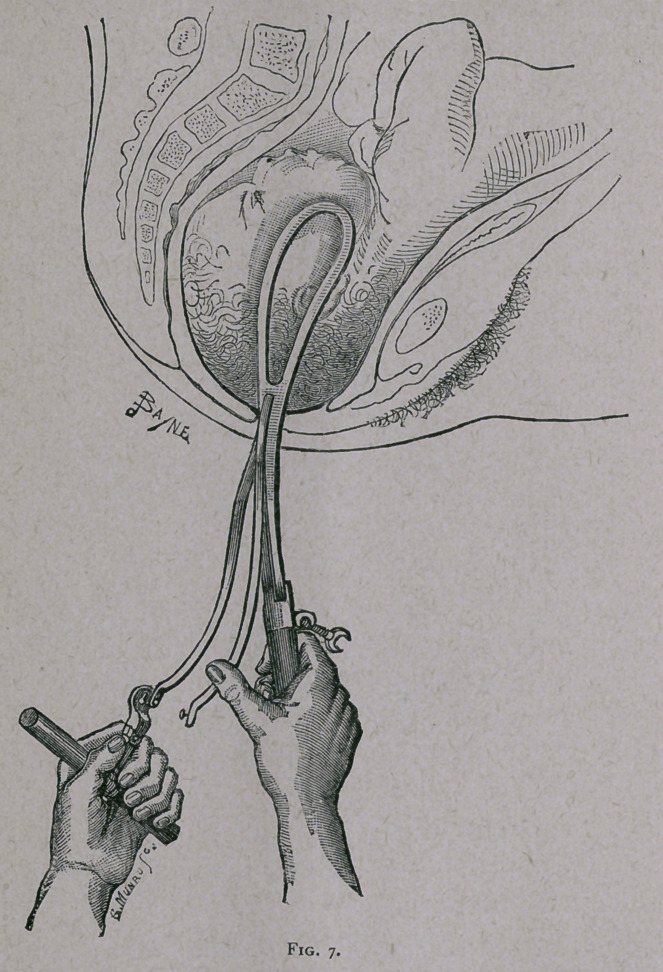# Axis-Traction Forceps

**Published:** 1884-01

**Authors:** 


### Selections.

### Axis-Traction Forceps.

Two years and a half have passed since I read a communication on Axis-Traction Forceps. I applied this designation to the forceps invented by M. Tarnier, or such modifications of them as I ventured to commend to the use of my fellow-practitioners. Their essential feature consists in their having traction-rods joined to the proximal end of the blades, and curving backwards towards a transverse bar which serves as the traction-handle of the instrument. The attachment of rods to the blades allows of direct traction on the head in their embrace. The backward compensation curve (perineal curve, it is sometimes called) of the rods allows of the traction by a curved instrument through a curved canal without loss of power, and without misdirection of force. The jointing of the rods allows the advancing head to move the application handles in the constantly-changing direction along which it is traveling; and the direction

Fig. 1.—Application of the first or left half of forceps, with traction-rod and traction-handle permanently attached.

of the application handles thus furnishes the operator with an unerring index to the proper line of traction.

Dr. Albert Smith alleges that the Tarnier instrument is only a resuscitation of the disused apparatus of Hermann of Berne. Hermann saw and indicated very forcibly the difficulty of extracting the head with a curved instrument through a curved canal. He felt the necessity of correcting the loss and misdirection of the power that results from traction through the handles, by making pressure in the neighborhood of the lock from the front (above, if the woman be on her back), or by

FIG. 2.—Introduction and adaptation of the first or left half of axis-traction forceps. 1 shows the traction-rod pushed in front of the shank during the process of introduction. 2 shows the traction-rod restored to its position when the blade has been adapted.

dragging on the lock from behind (below, if the woman be in the dorsal decubitus). To correct this loss and misdirection of power, one practitioner would rest the end of the long handles of the forceps on his shoulder while making traction with both hands clasped round the lock. Another would drag on the handles, and press with his knee upon the lock. I have seen an obstetrician who had committed the traction to two assistants pulling by the hooks at the handle-ends, himself with his hands clasped round the lock close to the vulva, pulling in a backward direction. I remember assisting Dr. Kirk nearly thirty years

FIG 3.—Commencement of introduction of the second or right half of axis-traction forceps, showing the traction-rod pushed forward past the shank.

ago, in a difficult case, where two of us made traction on the handles and on a towel tied round them, while he manipulated the lock and shanks so as to press back the head into the hollow of the sacrum. Now Hermann's contrivance simply consisted in applying an instrument like a two-pronged fork, with a transverse bar for a handle, into two depressions close to the forceps' lock. The fork is so contrived that it may be applied either from the anterior (upper or pubic) aspect, so as to make backward pressure while traction is made through the handles, or from the posterior aspect closer to the blades, so as to make backward traction to correct the loss and misdirection of force applied to the handles. But this ingenious attempt of

FIG. 4.—Second or right half of axis-traction forceps carried further on in the hollow of the sacrum, and moving toward the side of the fœtal head.

Hermann to render more precise the point at which the corrective pressure is applied, has nothing in common—and I wish broadly and again to emphasize my allegation—with the Tarnier traction-rods, which make traction, I repeat, directly on the head-embracing blades; which have a compensation curve allowing of direct traction in the axis through which, at any given moment, the centre of the head should move; and which are guided in the course of their action by the constant change in the direction of the application-handles—change permitted and produced by the loose jointing of the traction-rods to the heels of their respective blades. Dr. Smith seems to think that Hermann's contrivance was lost sight of, only because he had

FIG. 5.—Mode of locking the blades after the traction-rods have been thrown back to their proper position.

not the opportunity of proclaiming its value from a professional chair. He seems not to know that not only was Hermann Professor of Midwifery in the University of Berne—he enjoyed also the Persian blessing of being succeeded in his office by his son. No; his suggestion fell dead, because, as Dr. Smith's paper shows, its object can be effected just about as well or as ill with the unarmed hands. From Dr. Smith's own drawings we can see him attempting to avoid loss of power and misdirection of it by clasping his hand over the neighborhood of the lock, and making backward pressure with the ulnar edge, while the thumb pushes the extremities of the handles upward and forward; and Hermann made the same attempt, by pulling with one hand on

FIG. 6.—Locking-plate and joints of the traction-handle (full size). *a*, Joint for rotation of traction-bar; *b*, Joint for lateral movement; *c*, Opening and slot for locking of right tract on-rod; *d*, Left traction-rod permanently jointed to locking-pl te.

the handles, and pressing with the other, by means of his fork, on the lock.

There is more force in the allegation that Tarnier's forceps is the same in its principle of action as the forceps with recurved handles of Hubert or Aveling. Hubert and Aveling have both, and I believe independently, demonstrated the impossibility of making correct traction with forceps having curved blades and straight handles. They have both, accordingly, advocated the use of instruments made with a compensation or perineal curve on the handles. In this Tarnier forceps precisely corresponds

Fig. 7.

with such S-shaped forceps. The compensation curve on the traction-rods, where they approach the traction handle, has precisely the same effect as the curve in the handles of the scientifically constructed forceps of Hubert and Aveling—the effect, namely, of allowing direct traction in the axis of the pelvis without loss or misdirection of effort. But while the Tarnier forceps has the correct axis-traction curves in common with both these forceps, it has, in addition, the distinctive feature of having the traction-rods (as I have already said more than once) so jointed to the blades that the movements impressed on the application handles gave a constant guide to the operator as to the direction in which we ought to pull. Now this double property of the Tarnier forceps—of, *first*, giving power of correct traction, and, *second*, giving a guide to the proper direction—puts the obstetrician who uses it at a great advantage over the operator who has only an ordinarily curved instrument in his hand. We have used it in all imaginable cases, and have had recourse to the various manipulations recommended with the view of obviating the loss and misdirection of power that their construction involves. In the simple cases—and these, to be sure, constitute the majority of forceps cases—we have accomplished the delivery of the child to our perfect satisfaction, and without the expenditure of much strength or skill. But cases have sometimes met us that tried all our strength and taxed all our skill, that sometimes baffled us completely, or were terminated by the extraction of a damaged child from a damaged mother. And we find the axis-traction forceps effecting delivery with more safety to the woman and child. Let who will continue to use ordinary curved forceps, an obstetrician who has used the Tarnier forceps in a few test cases will no more think of reverting to the other than a man who can afford to keep a carriage will continue to practice as a peripatetic. He may use the defective instrument occasionally to keep muscle and mind in exercise, or because the case is so easy that it can be finished with anything, but in the general run of his work, and in all his difficult cases, the axis-traction forceps becomes for him a valued necessity.

Objections have been offered to the Tarnier forceps in regard to its construction. It is a misfortune that in his earliest models Tarnier had the compensation curve not only on the tractionrods, but on the application-handles. This curve in the handles was to my own mind one of its drawbacks. When we have become familiarized with the introduction of curved blades with straight handles, we feel as if we had to learn all our lesson over again when we begin to use an instrument with recurved handles, even of the simplest form, such as Hubert's or Aveling's. Tarnier has quite done away with the curve in the shank and handles, retaining only the curve of the traction-rods.

Objection continues to be made to the use of the fixation screw, which is supposed likely to cause and keep up too forcible compression of the head of the child, as if it could impart to the forceps something of the character of the cephalotribe. But it is important to remember that whatever the kind of forceps employed, the head is inevitably compressed. In the testing cases the operator who pulls on the head by grasping the ordinary handles, compresses the head with a force which he loses all power of estimating, and which perhaps he only realizes when he sees it imprinted, as it has been times without number, on the scalp or even skull of the infant. The use of a fixation screw enables the operator to compress the head to a degree which his unstrained muscular sense enables him to appreciate with great precision. He has only to fix the screw at the point where he feels that he has obtained a grasp of the head that is sufficient to hold it fast and that is safe in its amount of compression. It thus, as Prof. Howard has clearly recognized and fittingly stated, comes to be a "regulation screw," preventing the justly-dreaded danger of over-compression of head.

To my own axis-traction forceps the objection has been offered by Dr. Barnes that the instrument is too small. I have never yet met with a case where the model I exhibited to the Society did not suffice to reach the head and extract it even when the head lay above the brim. But I know no reason why the forceps should not be made as long as those of Dr. Barnes, at least as regards the blades and shanks. The forceps I have recently used accordingly measures nine inches from the lock to the tip of the blades. Besides thus increasing the length of the forceps, I have, at the suggestion of Dr. Hart, made a slight change in the flattened extremities of the rods to which the knobs are attached. It was found that the head of one of those knobs or buttons was liable to break, because the rods met the locking-plate at an angle. But by making the flattened ends of the rods pass at an angle from the stem and lie parallel to the locking-plate, all risk of such accident is obviated. Dr. Lyon objected that the traction-rods were not of sufficient strength. This difficulty also it would be easy to overcome. But I have tested the rods that I have had in use, and find that a weight of 200 lbs. does not strain them; and I do not suppose that in my most difficult case I have found it necessary to apply nearly half that force. [After describing several other forceps and improvements suggested to be made on the Tarnier instrument, the writer continues].

Every effort to improve the axis-traction forceps is a witness to the value of the principle on which they are constructed, and M. Tarnier, who has repeatedly modified the form of his own forceps, has shown no jealousy of the proposed modifications. Doubtless, changes will yet be suggested which will be found to render the instrument more manageable without compromising its utility. I can easily believe that in the process of evolution the application-handles will become lighter and less, and the locking-plate more simple and at the same time more secure. But whatever changes may be effected in the construction of the forceps, it seems to me that the perfect instrument must, *first*, have its traction-rods permanently attached each to its own blade. It is no advantage, but a drawback, to have the traction-rods so contrived that they may be used in one case and not in another. No operator who has the option of making axis-traction should ever expose himself to the temptation of pulling by the application-handles. I began myself with the error of fitting removable traction-rods to our British forceps, but quickly learned that the rods were a necessity for every case, and must be permanently attached to their respective blades. I think, even, that there will be no loose or separable part of the instrument, as in Tarnier's and Lusk's models, when the instrument is perfected.

This leads me to remark, *second*, that the traction-rods must be so contrived as to be capable of being easily locked and unlocked. Perhaps, for example, instead of having them meet in a locking-plate, as in my model, the traction-handle might be attached to the left rod by a ring, into which the right rod could be hooked. Then, *thirdly*, the fixation screw must be applied close to the lock, and be capable of easy manipulation, because, after each tractile effort it is well to relax it, and it must again be fixed before renewing the effort.

And now I will try to show you that the introduction and adaptation of forceps furnished with permanently attached traction-rods offer no greater difficulty than in the use of our ordinary forceps.

The left half of the instrument is, as usual, to be first introduced. This has the traction-bar or handle attached to its traction-rod; and if the rod be pushed in front of the shank it offers no difficulty to the manipulator. The handle should be held in the left hand, and guided into the canal with the fingers of the right, as seen in Fig. 1. The blade having been passed according to the usual rules, and adapted to the head, the traction-rod is pushed back to its ordinary position (see Fig. 2). The traction-bar is loosely jointed and swings freely about without at any time interfering with the passage of the second blade. When the patient is placed, as she commonly is with us, on her left side, it is not even necessary to take the precaution of pushing the left traction-rod in front of the shank. But in introducing the right half of the instrument, it is in every case necessary to push forward the traction-rod (see Fig. 3). If it be left swinging backwards it is apt to get entangled in the left half of the lock. This blade (held as usual by the right hand, and guided with the left) having been passed first backwards towards the hollow of the sacrum, and then round so as to be applied to the side of the child's head (see Fig. 4), the operator pushes back the traction-rod, and proceeds to lock the instrument in the usual way.

I was at one time afraid that in cutting off the German traction-hooks or shoulders we should be deprived of the power of easily adjusting and locking the blades, which we obtained by placing the thumbs on these lateral projections in our old familiar forceps. But I find that the projections of the fixation screw on the anterior surface afford the same facility. If, therefore, after the traction-rods are in their proper place, we grasp each handle with its own hand, and place the thumbs on the two parts respectively of the fixation screw (see Fig. 5), we obtain a purchase for moving the blade in any direction so as to secure their proper adaptation and their easy locking.

So far the application of axis-traction forceps corresponds to the application of the ordinary instrument, but for the necessity of shifting the position of one or both of the traction-rods. But now, further, we proceed to lock the loose right traction-rod by passing its knob or button into the slot in the locking-plates (see Fig. 6).

This manœuvre is not difficult, and is done in a second or two after we have practiced it for a few times either on the phantom or when simply holding the forceps in the two hands. The locking is effected by laying hold of the application-handles with the right hand and the traction-handle with the left. (See Fig. 7.) The thumb of the right hand lays hold of the free extremity of the right traction-rod, the forefinger of the left hand brings the locking-plate into relation with it, and so the locking is effected.

When an obstetrician has taken the trouble to apply to the head of the child a pair of axis-traction forceps, he finds himself more than compensated by the additional power he finds himself able to employ in the difficult cases, the delicacy of touch with which he feels that he can manage these cases, and the certainty with which he can pull in the pelvic axis in every case. Before applying traction, however, he grasps the application handles until he feels that he has got hold of the head so firm that the blades will not slip, yet not so forcible as to produce injurious pressure. At this point he secures the fixation screw. He then proceeds to make traction with one or both hands, grasping the transverse bar or traction-handle. In the great majority of cases the traction is made with the right hand alone, the left being free to watch the progress of the head. and guard the perineum. Traction is made as usual during the pains, and when no pains are present, at intervals. After each tractile effort the fixation screw may be slackened, to be fixed again before the next pull. The most important rule to be observed in using axis-traction forceps is to keep the traction-rods parallel with the shanks. When the rods are parallel with the shanks, the cord of the pelvic curve of the blades runs in a straight line to the centre of the traction-bar, and hence traction on the bar carries the head along the axis of the pelvic plane occupied at the given moment by the fœtal head. As the head descends the application-handles must never be touched, and they can be seen to move more and more forwards until at last, as the head emerges from the outlet, they lie nearly parallel with the abdominal wall.

I have already said that the use of properly curved traction forceps is necessary in every part of the parturient canal. And now, with regard to the perineal part of the canal, I wish to remark especially that axis-traction forceps with jointed rods enables us to guide the head more accurately along it. We can feel more distinctly the amount of strain we put on the tissues, can more delicately prevent too rapid expulsion of the head during parturient effort and make traction when the effort is passing off, and can all the time be sure that the head is progressing in the safest direction. When the head is born the fixation screw is first relaxed and the right traction-rod unlocked, and then each blade can be slipped off in turn.

I may sum up these remarks in the following propositions: 1. The extraction of the fœtal head through any part of the curved parturient canal demands the use of a forceps having the pelvic curve (curve of Smellie and Levret). 2. Extraction with such an instrument cannot be made without loss of power and mis-direction of power, unless the handles have a compensation curve (perineal curve of Johnstone, Moralés, Hubert and Aveling). 3. The addition to the blades by a joint or hinge of compensationally curved traction-rods gives the possibility of correct axis-traction, while the change impressed on the direction of the fixed application-handles affords an index to the operator as to the line at which at any moment he ought to pull (axis-traction rods of Tarnier).—*Edinburgh Medical Journal, New York Medical Abstract.*

## Figures and Tables

**Fig. 1. f1:**
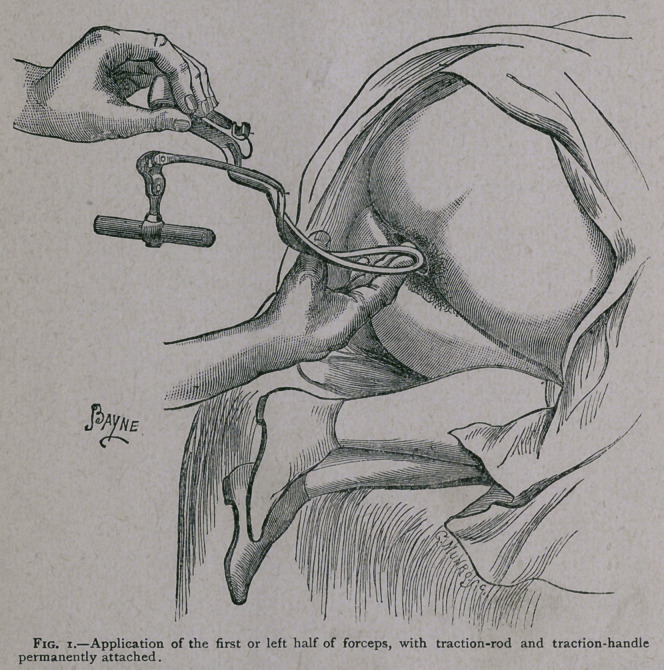


**Fig. 2. f2:**
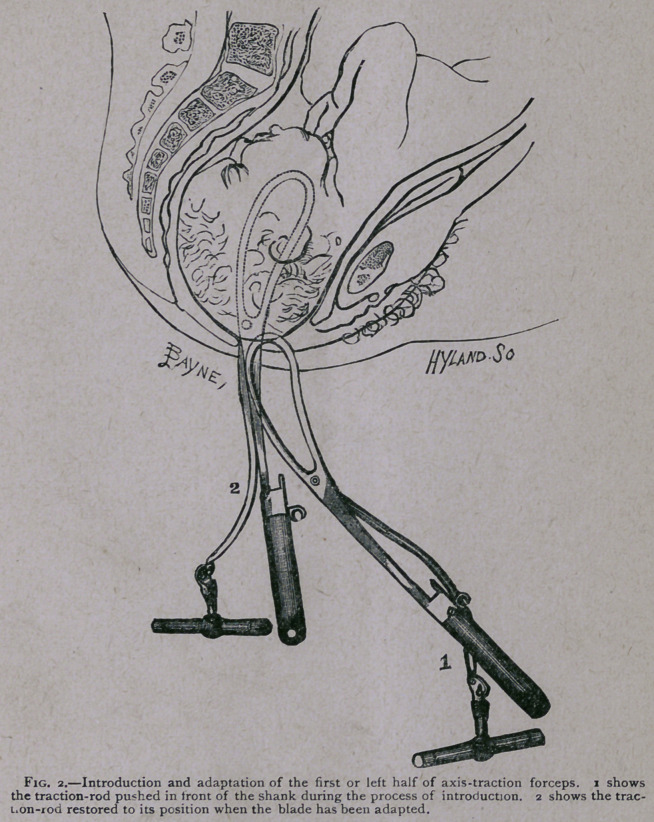


**Fig. 3. f3:**
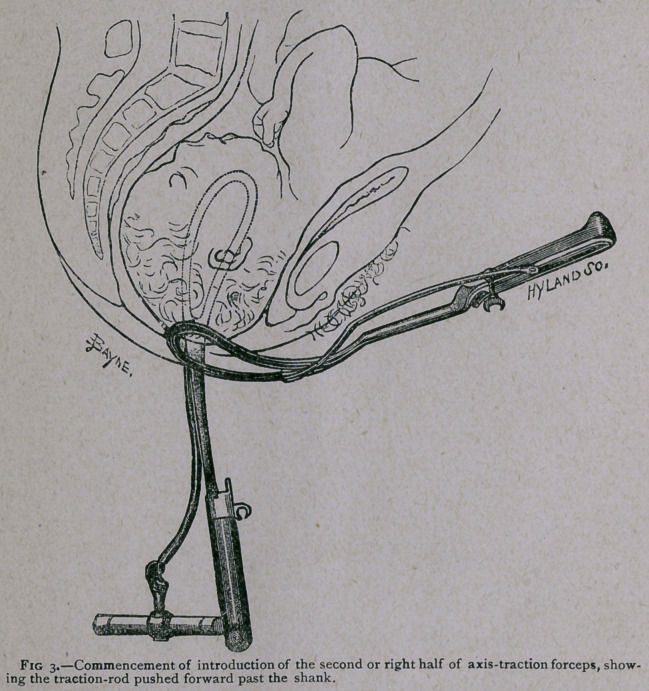


**Fig. 4. f4:**
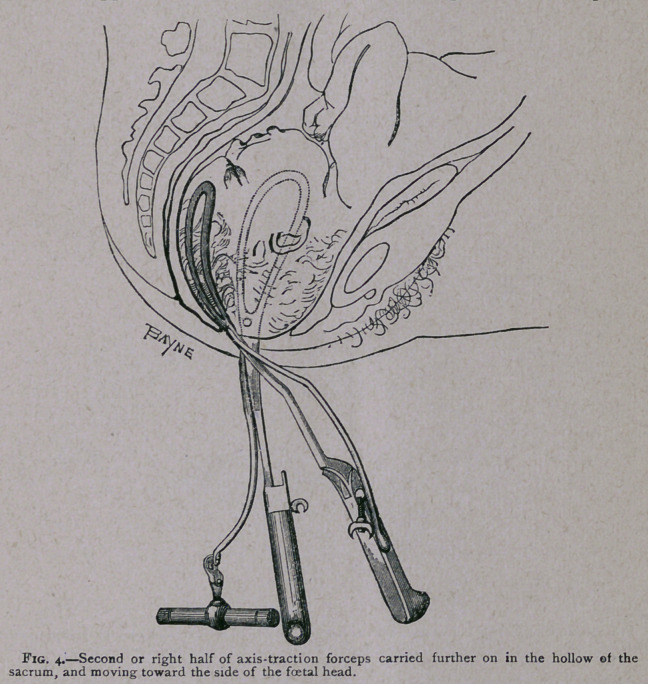


**Fig. 5. f5:**
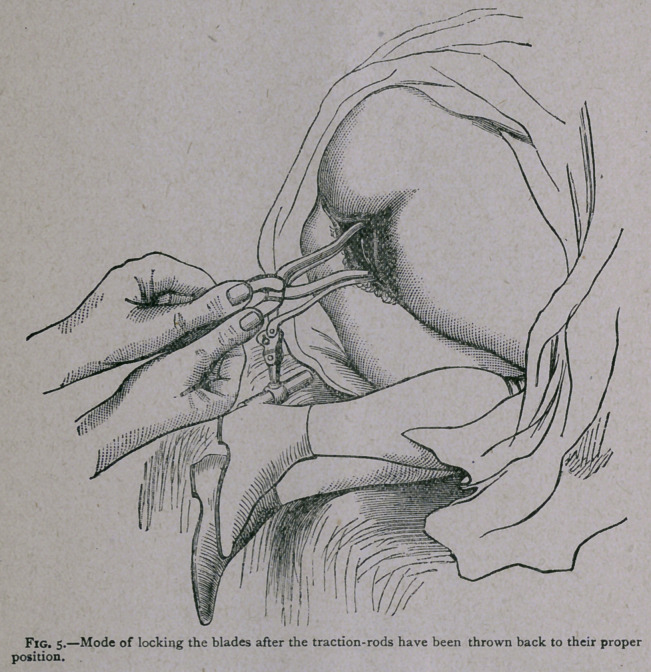


**Fig. 6. f6:**
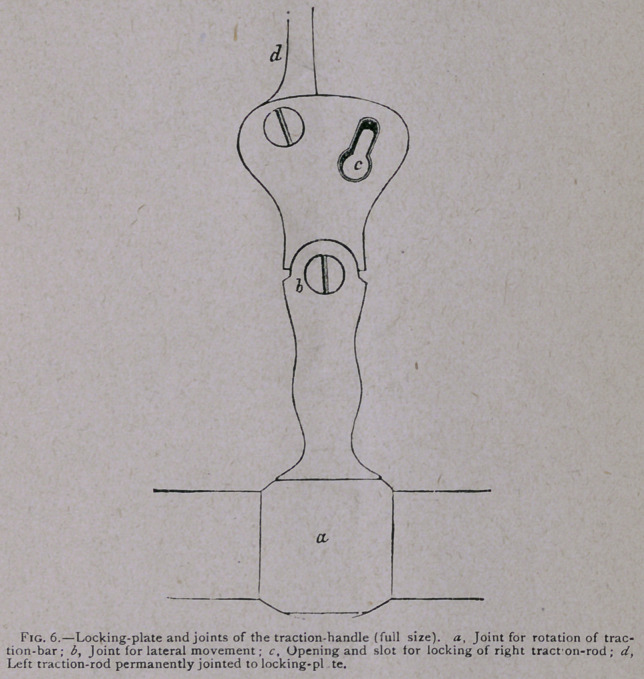


**Fig. 7. f7:**